# Differences in Vitreous Protein Profiles in Patients With Proliferative Diabetic Retinopathy Before and After Ranibizumab Treatment

**DOI:** 10.3389/fmed.2022.776855

**Published:** 2022-05-27

**Authors:** Xinping She, Chen Zou, Zhi Zheng

**Affiliations:** ^1^Shanghai Key Laboratory of Ocular Fundus Diseases, Department of Ophthalmology, Shanghai General Hospital, Shanghai Jiao Tong University School of Medicine, National Clinical Research Center for Eye Diseases, Shanghai Engineering Center for Visual Science and Photomedicine, Shanghai Engineering Center for Precise Diagnosis and Treatment of Eye Diseases, Shanghai, China; ^2^Eye Institute, Eye and ENT Hospital, Shanghai Medical College, Fudan University, Shanghai, China

**Keywords:** proliferative diabetic retinopathy, proteomics, ranibizumab, label-free, LC-MS/MS, PRM

## Abstract

Proliferative diabetic retinopathy (PDR) accounts for severe impact on vision, its mechanism is still poorly understood. To compare the differences of vitreous protein profiles in PDR patients before and after a complete anti-vascular endothelial growth factor (VEGF) loading dose with ranibizumab treatment. Twelve vitreous humor (VH) samples were collected from six PDR patients before (set as pre group) and after (set as post group) intravitreal injection of ranibizumab (IVR) treatment. LC–MS/MS and bioinformatics analysis were performed to identify differentially expressed proteins. Proteins were validated with targeted proteomics using parallel reaction monitoring (PRM) in a validation set consisting of samples from the above patients. A total of 2680 vitreous proteins were identified. Differentially expressed proteins were filtrated with fold change ≥2.0 (post group/ pre group protein abundance ratio ≥2 or ≤ 0.5) and *p*-value <0.05. 11 proteins were up-regulated and 17 proteins were down-regulated, while consistent presence/absence expression profile group contains one elevated protein and nine reduced proteins, among which seven proteins were identified as potential biomarkers for IVR treatment through PRM assays. Bioinformatics analysis indicated the up-regulated proteins were significantly enriched in “GnRH secretion” and “Circadian rhythm” signaling pathway. This report represents the first description of combined label-free quantitative proteomics and PRM analysis of targeted proteins for discovery of different proteins before and after IVR treatment in the same patient. IVR treatment may protect against PDR by promoting SPP1 expression through “GnRH secretion” and “Circadian rhythm” signaling pathway.

## Background

Diabetic retinopathy (DR) is the leading cause of blindness among working age people (1–4). The worldwide prevalence of DR has been estimated to be 34.6% in patients with diabetes, and the prevalence of vision-threatening DR, such as proliferative diabetic retinopathy (PDR) has been estimated to be 6.96% (5). PDR is the worst stage of DR, it may lead to devastating complications, such as vitreous hemorrhage or tractional retinal detachment.

Several studies have shown that vascular endothelial growth factor (VEGF) is a crucial causative factor of PDR (6, 7). Ranibizumab is a specific anti-VEGF drug, it is an engineered, humanized and recombinant antibody fragment binding closely to all VEGF-A isoforms. Preoperative intravitreal injection of ranibizumab (IVR) treatment significantly reduces the occurrence of intraoperative and postoperative complications (8). A meta-analysis of 14 randomized controlled trials indicated that anti-VEGF pretreatment before vitrectomy greatly facilitated surgery (9). Another network meta-analysis revealed that preoperative anti-VEGF pretreatment showed the best treatment effect (10). However, the molecular mechanism is not completely clear. We previously reported that preoperative IVR treatment in patients with severe PDR contributes to a decreased risk of postoperative neovascular glaucoma (11), and found further changes in vitreous protein profiles of PDR patients treated with and without IVR (12). While there have been no reports on the changes in vitreous humor (VH) protein profile before and after IVR treatment in the same patient. Taking the influence of individual differences into account, the VH samples of the same patient before and after IVR treatment were tested, then the identified differences can be considered to be entirely caused by IVR. This is a research topic worthy of further study. Thus, it is of interest to study differences of vitreous protein profile in PDR patients before and after ranibizumab treatment.

Proteomics have been widely applied for global analysis of proteins (13), and it has great value for studying the effects of DR (14, 15). Label-free quantification is a type of quantitative mass spectrometry method. This technology does not require expensive isotope labels as internal standards, but it improves the detection efficiency of low-abundance proteins and the accuracy of protein quantification. Using label-free quantification technology, the sample loading volume is small. In recent years, label-free quantification has been commonly applied for the study of DR (16–18). Parallel reaction monitoring (PRM) is an ion monitoring technology based on high-resolution and high-precision MS. Compared with western blotting and ELISA, it has higher sensitivity and higher resolution and unlike these other methods, it can be used for the simultaneous detection of multiple target proteins without the need for antibodies (19). Therefore, it is often used as a verification method.

In this study, 12 vitreous samples were collected from six PDR patients before and after IVR treatment, and label-free technology combined with PRM target validation was used to conduct proteomic analysis of and assess the VH samples. This study aimed to identify differences in vitreous protein profiles in patients with PDR before and after IVR treatment and to further reveal the potential therapeutic targets of ranibizumab in PDR patients.

## Materials and Methods

### Patients and Sample Collection

Six PDR patients who required vitrectomy were recruited from the department of ophthalmology, Shanghai General Hospital. The protocol was approved by the Research Ethics Committee of Shanghai General Hospital (Ethical approval number: 2021KY031). Signed informed consent was obtained from all patients, and the experimental procedures followed the tenets of the declaration of Helsinki. Patients' rights to privacy were protected in this study. All of the PDR participants were screened according to the expert consensus for the prevention and treatment of DR. Examinations were carried out by a professional ophthalmologist after pupil dilation. Clinical characteristics of the study population are shown in Table 1.

**Table 1:** Clinical characteristics of the patients.

**Patient**	**Gender**	**Age(years)**	**Diabetes course(years)**	**Surgical eye**	**Vision**	**IOP(mmHg)**
5	M	33	2	OS	HM	17.4
6	M	31	13	OS	0.4	16.2
7	M	46	13	OD	HM	12.3
8	F	60	5	OD	0.25	12.5
9	M	53	20	OD	0.01	16.5
10	F	27	8	OD	0.04	12.1

*IOP, intraocular pressure*.

The inclusion and exclusion criteria were as follows. Patients who met the diagnostic criteria of PDR with vitreous hemorrhage were eligible for inclusion in the study. Eyes that received laser or intraocular injection therapy within 3 months were excluded; patients with retinal vein occlusion, retinopathy of prematurity, sickle cell retinopathy, familial exudative retinopathy and other retinal vascular diseases were excluded.

Before IVR treatment, we used 25G vitreous cutter (Constellation; Alcon Instruments, Inc., Fort Worth, TX, USA) to collect 0.25–0.3 mL VH without any infusion, and these VH samples were used as the pre group. A portion of the VH was extracted to makes room for the injection of ranibizumab, and 0.5 mg/0.05 mL ranibizumab (Lucentis; Novartis Pharma Schweiz AG Inc., Schaffhauserstrasse 4332 Stein, Switzerland) was injected. Three days later, we used 25G vitreous cutter to collect 0.25–0.3 mL VH without any infusion before pars plana vitrectomy (PPV), and which were used as the post group. After obtaining the vitreous sample, we immediately performed a centrifugation. 0.25–0.3 mL of undiluted VH was centrifuged for 10 min at 4 °C and 15000 rpm; then the supernatant was stored in liquid nitrogen and analyzed later. Sample processing refers to the method in our previous publication (12).

### Sample Processing

VH samples were lysed according to the FASP procedure (20), and proteins were extracted by using buffer 1 (4% SDS, 100 mM Tris-HCl, 1 mM DTT; pH 7.6). The concentration of protein was quantified with the BCA Protein Assay Kit (Bio-Rad, USA). A filter-aided sample preparation procedure (20) was used for protein digestion. 200 μg of protein from each sample was added to 30 μL buffer 2 [4% SDS, 100 mM DTT, 150 mM Tris-HCl (pH 8.0)]. Protein suspensions were digested with 4 μg trypsin (Promega) in 40 μL 25 mM NH4HCO3 buffer overnight at 37°C. The peptides were desalted on C18 cartridges [Empore™ SPE Cartridges C18 (Sigma)], concentrated by vacuum centrifugation and reconstituted in 40 μL of 0.1% (v/v) formic acid. UV light spectral density at 280 nm was used to estimate the peptide content.

Label-free quantification analysis was performed on a trapping ion mobility mass spectrometer (Bruker, timsTOF™ Pro). The mass spectrometer was operated in positive ion mode. A Pierce high pH reversed-phase fractionation kit (Thermo Scientific) was used to fractionate samples into six fractions by increasing acetonitrile step-gradient elution according to the instructions. MS data were acquired using a data-dependent top 10 method by dynamically choosing the most abundant precursor ions from the survey scan (100–1700 m/z) for higher-energy C-trap dissociation fragmentation. The raw MS data for each sample were combined and searched using MaxQuant 1.5.3.17 software. Parameters and instructions are shown in Table 2.

**Table 2:** Maxquant identification and quantification parameter table.

**Item**	**Value**
Enzyme	Trypsin
Max missed cleavages	2
Main search	6 ppm
First search	20 ppm
MS/MS tolerance	20 ppm
Fixed modifications	Carbamidomethyl (C)
Variable modifications	Oxidation (M)
Database	Swissprot_Homo_sapiens_ 20395_20210106.fasta
Database pattern	Reverse
Include contaminants	TRUE
Protein FDR	≤ 0.01
Peptide FDR	≤ 0.01
Peptides used for protein quantification	Use razor and unique peptides
Time window (match between runs)	2 min
protein quantification	LFQ
min. ratio count	1

### Bioinformatics Analysis

Hierarchical clustering analysis was performed by using Cluster 3.0 and Java TreeView software. The protein sequences of the selected differentially expressed proteins were locally searched using NCBI BLAST and InterProScan to find homologous sequences. Gene Ontology (GO) terms were mapped, and the sequences were annotated using the software program Blast2GO (https://www.blast2go.com/). The GO annotation results were plotted by R scripts. Proteins were blasted against the online Kyoto Encyclopedia of Genes and Genomes (KEGG) database (http://geneontology.org/) to retrieve their KEGG orthology identifications. Enrichment analysis was performed based on Fisher's exact test. Protein–protein interactions (PPIs) were retrieved from the IntAct molecular interaction database using gene symbols or STRING software, and *P*-values < 0.05 were considered significant.

### Validation of Proteomic Analysis

To further verify the LC-MS/MS results, PRM analysis was performed for the same samples used in the MS discovery phase (*n* = 6 in both the post group and pre group) by using a high-resolution Q-Exactive HF mass spectrometer (Thermo Scientific, USA). The isotope relabeling peptide (PRTC:GLILVGGYGTR) was spiked in each sample and used as a standard internal reference. The original PRM files were analyzed using SKYLINE 3.5.0 software.

### Statistics

IBM SPSS 20.0 (SPSS, Inc., USA) and SAS (version 9.4) were used for statistical analysis. The Venn diagram was generated using an online tool developed by the Van de Peer Laboratory (Bioinformatics & Evolutionary Genomics). Comparisons among groups were conducted using paired sample *t-*test. Definition of proteins with present or absent expression was that, two or more times in one set of samples are not null values, and all the data in the other set are null values. Quantifiable proteins can be defined as more than half of the biological replicates have quantitative information. When screening differentially expressed proteins, the criterion of fold change (FC) >2 times or FC < 0.5 times, and *P-* value < 0.05 was applied.

## Results

### Identification and Quantification of Protein Profiles

**Figure 1:**
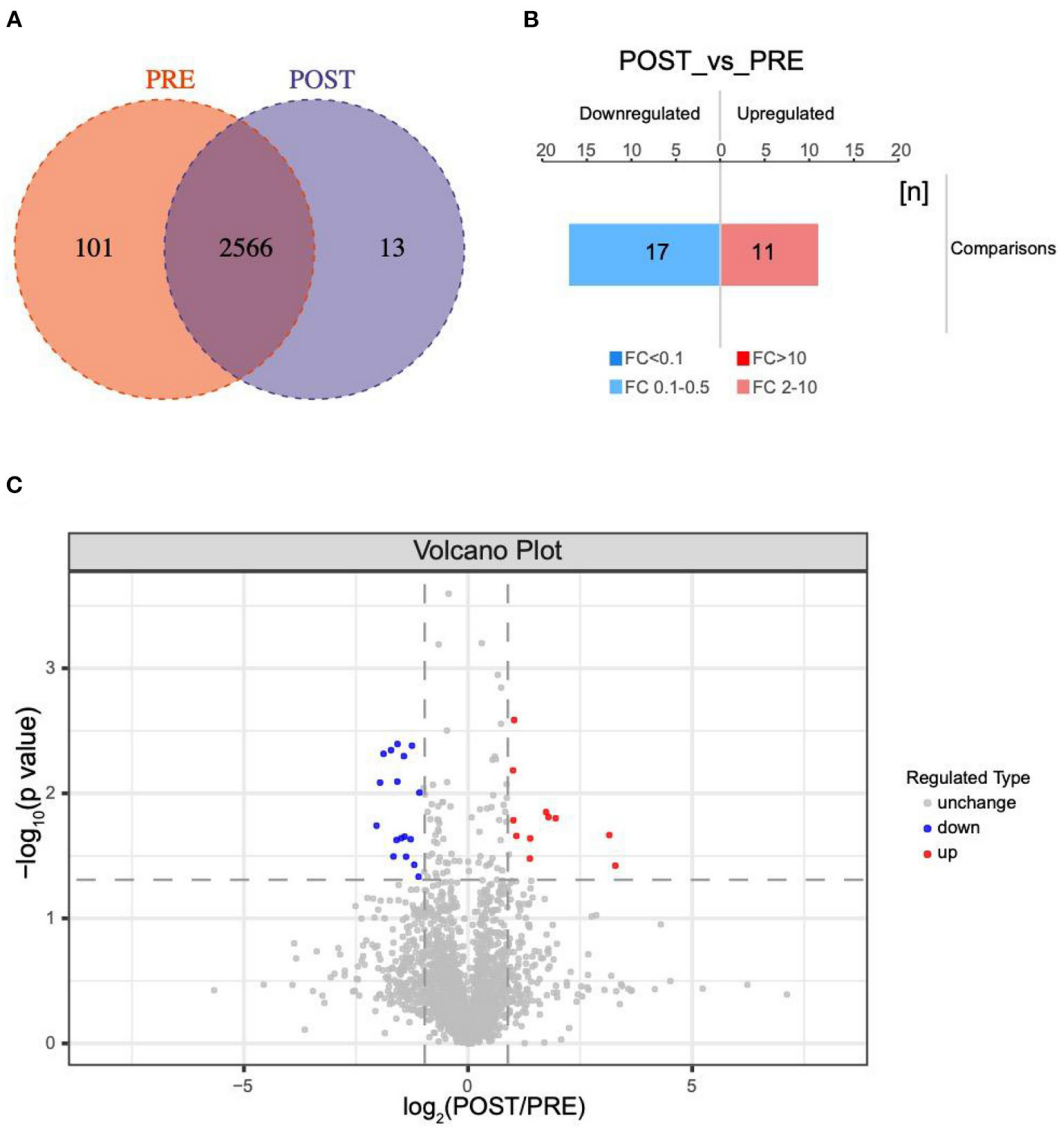
Changes in protein profiles before and after IVR treatment. **(A)** Venn diagram of differentially expressed proteins between the post group and pre group; **(B)** Histogram showing quantitative differences in protein expression between the post group and pre group. Significantly down-regulated proteins are marked in blue (FC < 0.5 and *P* < 0.05), and significantly up-regulated proteins are marked in red (FC > 2 and *P* < 0.05); **(C)** Volcano plot showing the significant differences in protein expression between the post group and pre group (*P* < 0.05).

A total of 2680 VH proteins were identified in this study (Supplementary Table S1). Among these proteins, 13 were found solely in the post group, 101 were found solely in the pre group, and the other 2566 proteins were found in both the pre group and post group (Figure 1A). Venn diagrams were used to analyze the overlap of proteins between the pre group and post group (Figures S1A,B).

A total of 38 proteins were differentially expressed in the post group compared with the pre group, including 11 up-regulated and 17 down-regulated, one only found in POST-group and nine exclusived to PRE-group (Figure 1B). Significantly down-regulated proteins are marked in blue FC < 0.5 and P < 0.05, while significantly up-regulated proteins are marked in red (FC > 2.0 and P < 0.05) in the volcano plot in Figure 1C. The database species used was Swissprot_Homo_sapiens_20395_20210106.fasta.

### GO Function Analysis of Differentially Expressed Proteins

**Figure 2:**
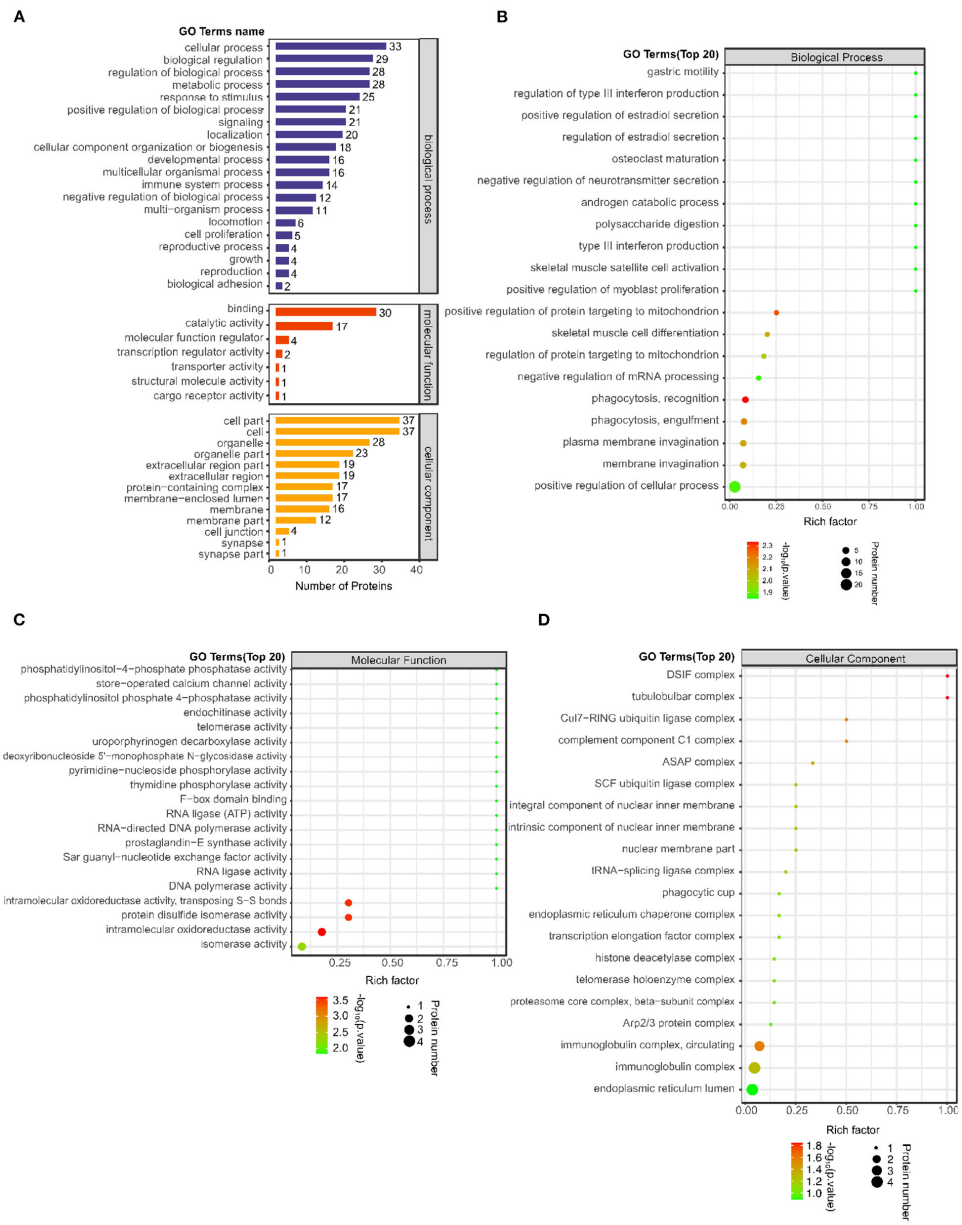
Number of differentially expressed proteins on the GO secondary function annotation level and Top 20 GO terms between the post IVR group and pre IVR group. **(A)** Graph of GO annotation of the differentially expressed proteins according to GO secondary function annotation. BPs, MFs and CCs are shown in blue, red and orange, respectively; **(B–D)** Bubble charts showing the results of enrichment analysis of BPs, MFs, and CCs for the differentially expressed proteins using Fisher's exact test (*P* < 0.05). The color represents the *P*-value (take -log10); the closer the color is to red, the smaller the *P*-value and the higher the significance of the enrichment of the corresponding.

A total of 1874 GO terms related to all 38 differentially expressed proteins were identified using Blast2GO software (Supplementary Table S2). Furthermore, the number of differentially expressed proteins was determined according to GO secondary function annotation. Among the GO secondary functions, 20 subcategories were in the biological process (BP) category, 7 were in the molecular function (MF) category and 13 were in the cellular component (CC) category. The top GO terms from each category were selected (Figure 2A). The predominant term in the BP category was “cellular process” (33 proteins), followed by “biological regulation” (29 proteins), “regulation of biological process” (28 proteins), “metabolic process” (28 proteins), “response to stimulus” (25 proteins), and “positive regulation of biological process” (21 proteins). The largest number of proteins were involved in the MF “binding” (30 proteins), followed by “catalytic activity” (17 proteins). The largest number of proteins were enriched in the CCs “cell part” (37 proteins) and “cell” (37 proteins), followed by “organelle” (28 proteins).

To reveal the overall functional enrichment characteristics of all differentially expressed proteins and to identify the most important significantly enriched GO terms, Fisher's exact test (*P* < 0.05) was applied to perform enrichment analysis of the differentially expressed proteins. The BP term that exhibited the most significant change in enrichment was “phagocytosis, recognition,” the MF term that exhibited the most significant change in enrichment was “intramolecular oxidoreductase activity,” and the CC term that exhibited the most significant change in enrichment was “DSIF complex” (Fig 2B–D). The main proteins involved were immunoglobulin heavy variable 3–23 (IGHV3-23), RNA-splicing ligase RtcB homolog (RTCB), osteopontin (SPP1), thymidine phosphorylase (TYMP), proactivator polypeptide-like 1 (PSAPL1), puromycin-sensitive aminopeptidase (NPEPPS), and complement C1q subcomponent subunit A (C1QA).

### KEGG Pathway Analysis

All 38 differentially expressed proteins were blasted against the online KEGG database and were subsequently mapped to KEGG pathways.

**Figure 3:**
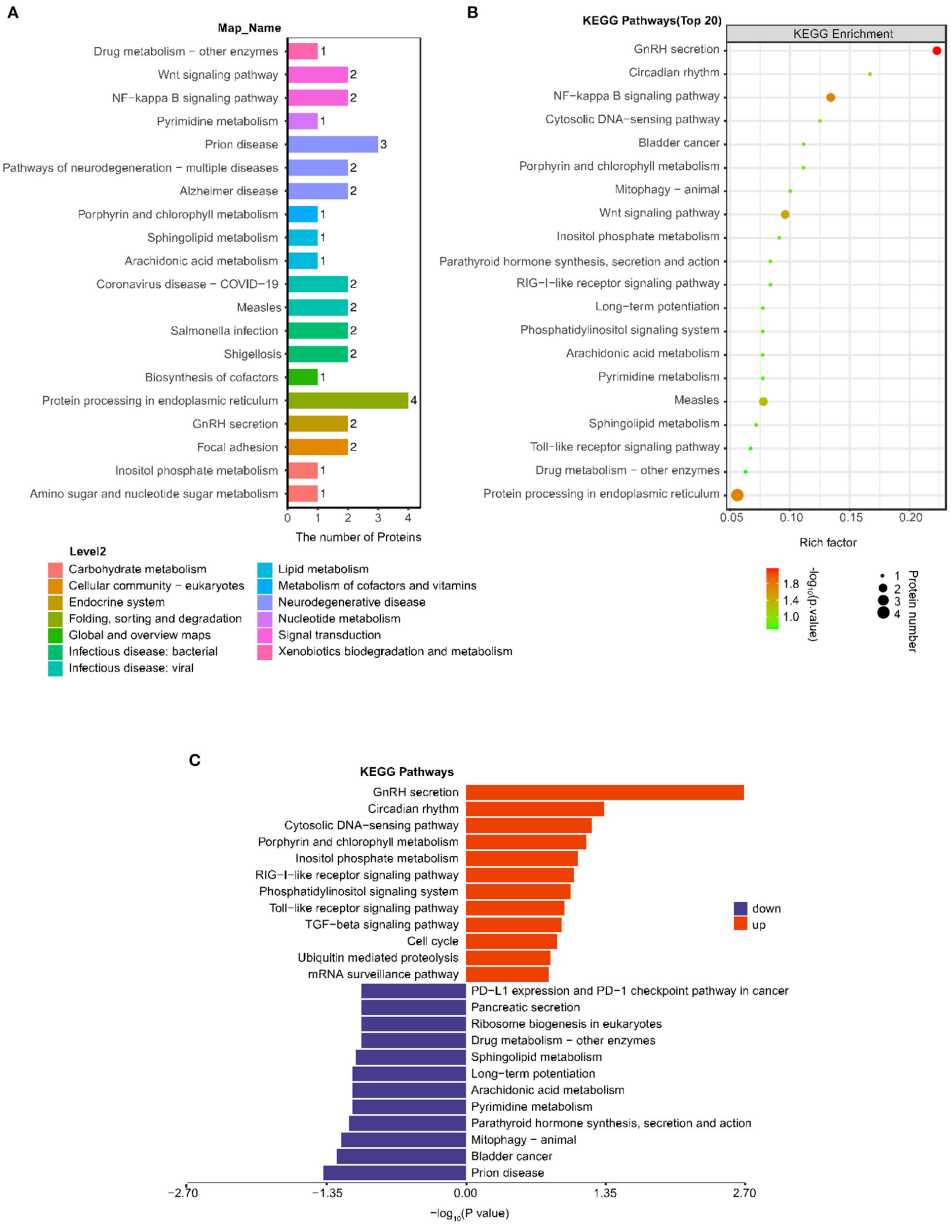
Analysis of changes in KEGG pathway between the post IVR group and pre IVR group. **(A)** Histogram showing the top 20 KEGG pathways in which the differentially expressed proteins between the post and pre group; **(B)** Bubble chart showing the enrichment of the top 20 KEGG pathways, as determined by Fisher's exact test (*P* < 0.05); **(C)** Pathways in which the up-regulated and down-regulated differentially expressed proteins were enriched.

As shown in Figure 3A, the most notable pathway was “Protein processing in endoplasmic reticulum” (four proteins), followed by “Prion disease” (three proteins), “GnRH secretion”(two proteins), “NF-kappa B signaling pathway” (two proteins), and “Wnt signaling pathway” (two proteins). Furthermore, we used Fisher's exact test (*P* < 0.05) to perform KEGG pathway enrichment analysis of the 38 differentially expressed proteins. The results showed that “GnRH secretion” exhibited the most significant change in enrichment followed by “NF-kappa B signaling pathway” and “Protein processing in endoplasmic reticulum” (Figure 3B).

To better investigate the significance of the differences in the pathways for which the differentially expressed proteins were enriched, we performed KEGG pathway and pathway enrichment analyses of the up-regulated and down-regulated proteins separately (Figure 3C). The down-regulated proteins were significantly enriched in “Prion disease” (three proteins, *P* = 0.0417), while the up-regulated proteins were significantly enriched in “GnRH secretion” (two proteins, *P* = 0.0020), “Circadian rhythm” (one protein, *P* = 0.0461).

### Protein Interaction Network Analysis

**Figure 4:**
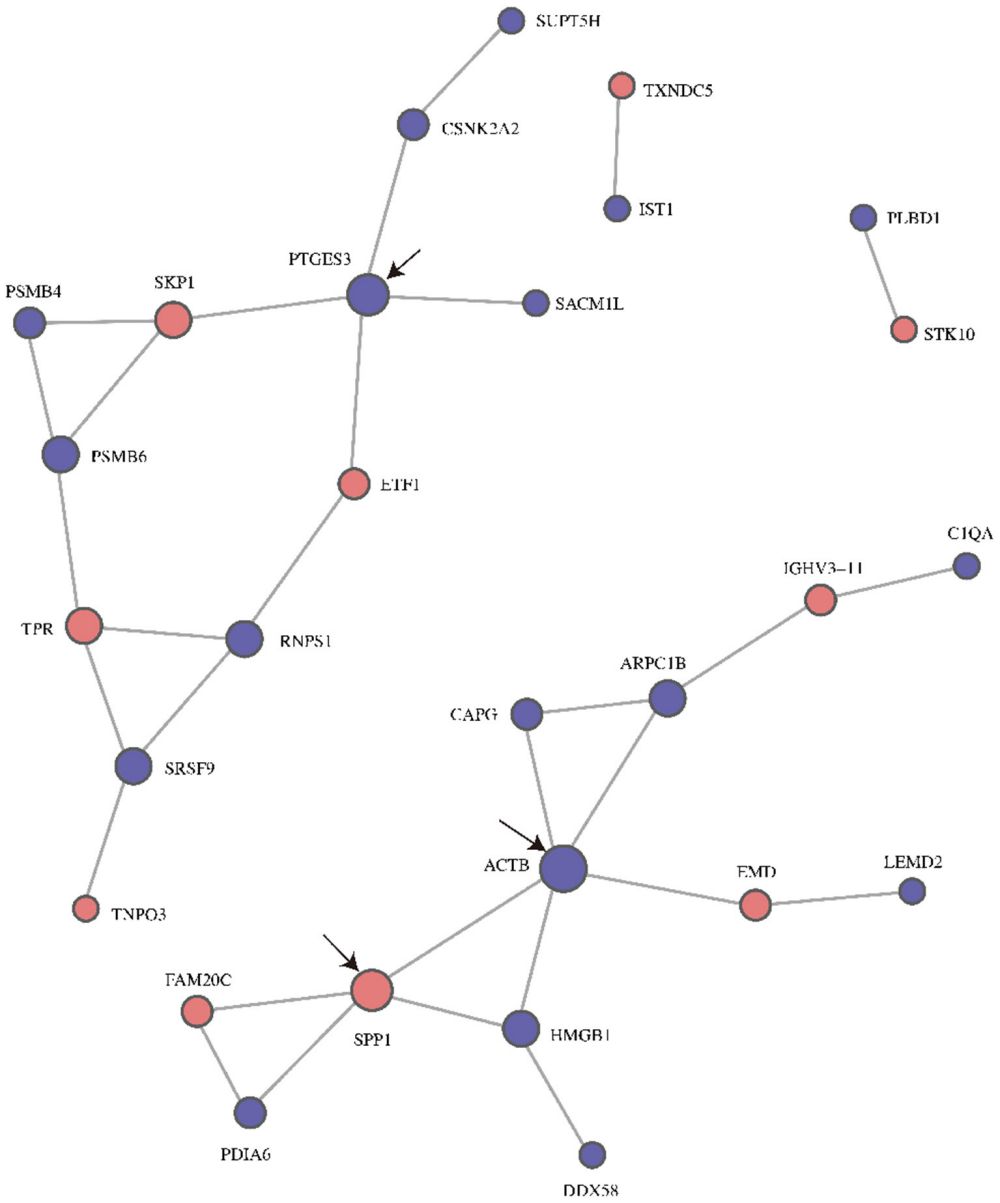
PPI network diagrams for differentially expressed proteins post- and pre- ranibizumab intraocular injection. The circled nodes in the figure represent down-regulation (blue) and up-regulation (red), the line represents the interaction between protein and protein. the black arrows marked the three proteins with highest connectivity. PPI, Protein-Protein Interaction; ACTB, Actin, cytoplasmic 1; SPP1, Osteopontin; PTGES3, Prostaglandin E synthase 3.

The PPI network diagram showed that 28 of the 38 differentially expressed proteins were involved in the interactive network (Figure 4). According to intergroup analysis and comparison, the proteins ACTB (Actin, cytoplasmic 1), SPP1 and PTGES3 (Prostaglandin E synthase 3) had larger circles than the other proteins, indicating that they might be the key points that affect the metabolic or signal transduction pathways of the entire system.

### Verification of Candidate Proteins by PRM

**Figure 5:**
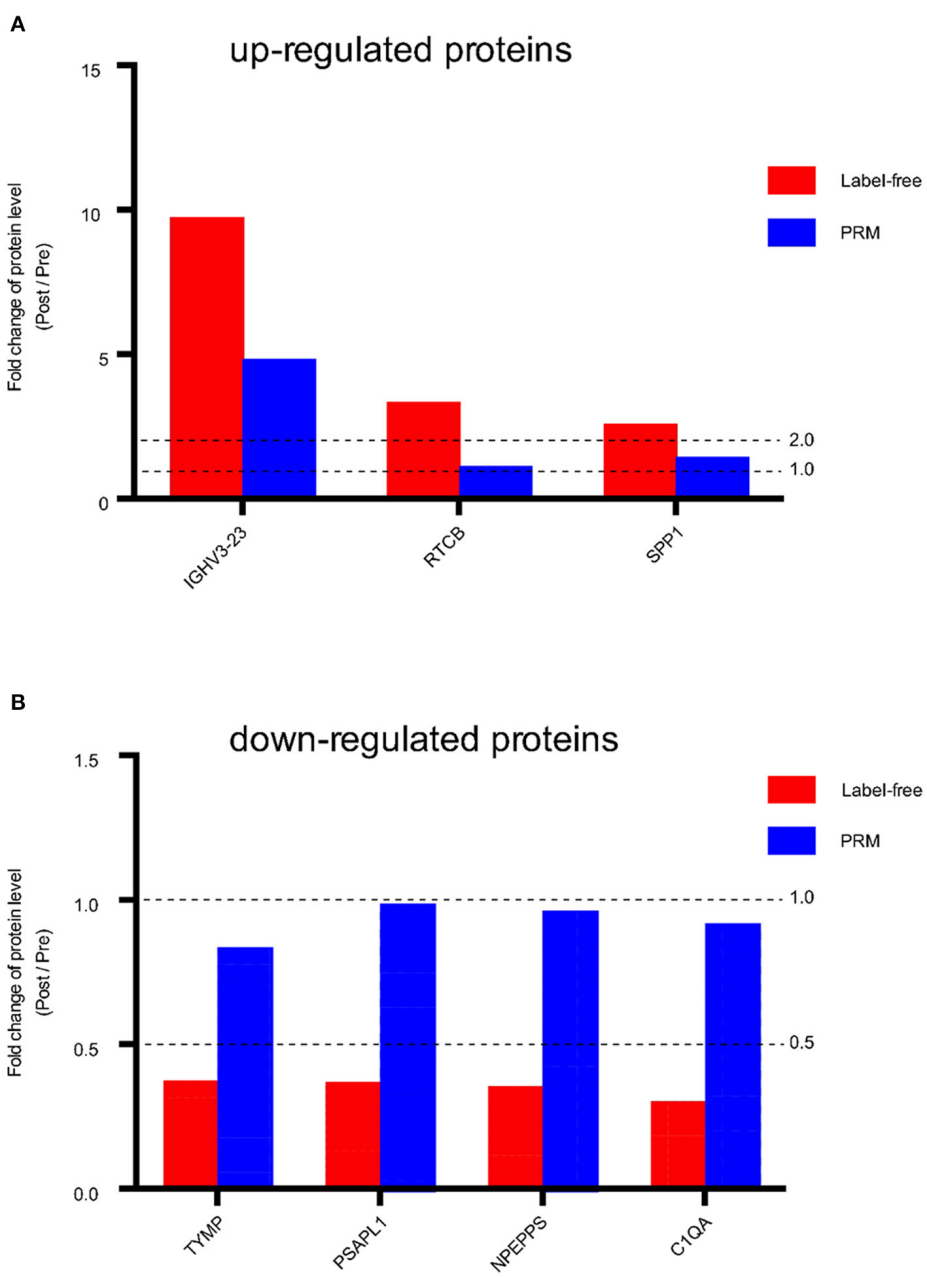
Fold change of protein level (Post / Pre) in Label-free and PRM verification. **(A)** Up-regulated proteins between the post IVR group and pre IVR group. **(B)** Down-regulated proteins between the post IVR group and pre IVR group. Fold change (FC) >2 times or FC < 0.5 times, and *P*-value < 0.05.

Seven proteins that showed significant changes in expression, including IGHV3-23, RTCB, SPP1, TYMP, PSAPL1, NPEPPS, C1QA, were examined by PRM. These proteins had larger FC values and are associated with potentially important biological functions related to angiogenesis, proliferation, and fibrosis. The expression of three of the proteins (IGHV3-23, RTCB, SPP1) was up-regulated in the post group compared with the pre group, and the expression of four proteins (TYMP, PSAPL1, NPEPPS, C1QA) was down-regulated in the post group compared with the pre group. We found that the overall trends of the label-free quantification and PRM results were consistent (Figure 5, Supplementary Table S4). The consistency of the PRM and label-free quantification results indicated the reliability of our proteomic data. Information such as peptide, precursor Mz, fragment ion, areas and other original data are shown in Supplementary Table S3. Targeted peptide Skyline analysis results were shown in Supplementary Figure S2.

## Discussion

In our present study, a total of 38 significant differentially expressed proteins were identified in the VH of PDR patients collected before and after IVR treatment. In our previous study, we identified differentially expressed proteins between PDR patients who received anti-VEGF therapy and those who did not (12, 21). However, in those two studies, the treated samples and untreated samples were collected from different patients. The design of our study is a paired sample, which compares the preoperative and postoperative samples of the same person.

Bioinformatics analysis indicated that the most significantly enriched BP was “phagocytosis, recognition.” The signaling pathway that most significantly enriched was “protein processing in endoplasmic reticulum.”

We further analyzed the up-regulated proteins and down-regulated proteins among the 38 differentially expressed proteins separately using the database. The up-regulated proteins were significantly enriched in “GnRH secretion” and “Circadian rhythm” signaling pathway.

Among the differentially expressed proteins identified by LC-MS/MS, we were particularly interested in seven proteins (IGHV3-23, RTCB, SPP1, TYMP, PSAPL1, NPEPPS, C1QA). These proteins play critical roles in angiogenesis, proliferation, and fibrosis, which are all closely associated with the development of PDR. However, the role of these proteins in the pathogenesis of DR is unclear. In our study, we verified the differential expression of these candidate proteins by PRM, and the results were generally consistent with those obtained by LC–MS/MS. These results suggest that these proteins may be important in the pathogenesis of DR.

IGHV3-23 belongs to a group of approximately 40 functional variable (V) genes in the immunoglobulin heavy chain locus on chromosome 14, and the variable domain participates in antigen recognition. IGHV3-23 may play a pathologically relevant role in the occurrence or progression of thymic MALT lymphoma (22). In chronic lymphocytic leukemia, IGHV3 gene is being highly utilized and with high mutational load, it has been shown to that display a bad prognosis (23). A relatively large Taiwanese cohort of chronic lymphocytic leukemia showed the most frequent usage of IGHV3-23 gene (24). In COVID-19 patients, IGHV3-23 was over-represented and was identified as novel B-cell-receptor (25). RTCB is a catalytic subunit of the tRNA-splicing ligase complex that acts as an RNA ligase with broad substrate specificity and may act on RNAs. Recent studies on Parkinson's disease have shown that RTCB-1 can play a neuroprotective effect by splicing XBP-1 mRNA (26). Another instance of a nonsplicing function for a tRNA processing factor is the discovery that, following axonal injury, RtcB mutants in C. elegans exhibit axon regeneration times that are faster than those of wild-type nematodes (27). This role for RtcB depends on its ligase activity and appears to be specific to neurons. SPP1 (synonym osteopontin), is a glycosylated protein. It is the main adhesion and chemotactic factor for vascular cells. As an angiogenic and fibrogenic factor, SPP1 has been reported to be expressed in patients with DR (28). Plasma SPP1 levels are associated with the presence and severity of DR, suggesting that SPP1 may be a potential biomarker for DR. Ang II upregulated SPP1 expression in adult rat cardiac fibroblasts by ROS-mediated activation of ERK1/2 and JNK pathways (29). A recent study demonstrated that S100A4 induces NF-κB-dependent expressions and secretions of SPP1 in osteosarcoma cell lines (30). These findings suggest that SPP1 may be a molecular mechanism related to S100A4 signaling. The increases in IGHV3-23, RTCB, and SPP1 expression indicated that ranibizumab may play an immune-activating and neuroprotective role in PDR patients.

TYMP has a role in inducing chemotaxis of ECs and angiogenesis. Through its enzymatic activity, TYMP produces 2-deoxy-d-ribose-1-phosphate from thymidine; subsequent hydrolysis generates 2-deoxy-d-ribose, which is the molecule that exerts chemotactic and angiogenic effects (31). A clinical study identified PSAPL1 genes to be enriched in the patients with face and neck atopic dermatitis(AD), suggesting that innate immune system is potentially associated with the pathophysiology of face and neck AD (32). PSAPL1 was associated with breast cancer grade and involved in the epithelial cell differentiation pathways and the sphingolipid metabolic process (33). Protein PSAPL1 was diferent in non-lesional and lesional samples compared to healthy skin and might represent proteins that contribute to maintaining the non-lesional state (34).

NPEPPS is involved in proteolytic events essential for cell growth and viability. It is required for the proliferation of myoblasts in the growth phase (35). C1QA associates with the proenzymes C1r and C1s to yield C1, the first component of the serum complement system. A study identified C1qA as a novel AGE-binding protein in human serum and found that it participates in stimulating the classical complement pathway (36). The decrease in the expression of these four proteins, TYMP, PSAPL1, NPEPPS, C1QA, indicates that ranibizumab may have a protective effect in PDR patients by reducing angiogenesis, inhibiting cell proliferation, inhibiting complement activation, etc.

The choice of ranibizumab is based on our previous research (12). Ranibizumab inhibits all isoforms of VEGF-A to block the activation of the VEGFR-1 and VEGFR-2 receptors, which prevents subsequent neovascularization due to receptor activation (37). Compared with bevacizumab, ranibizumab has a higher VEGF165 binding affinity (38) and achieves robust DR regression. Ranibizumab is a chimeric molecule that includes a nonbinding human sequence which makes it less antigenic in primates and a high affinity epitope that binds to VEGF-A (39). Ranibizumab appears to have some benefits in terms of systemic adverse events than other anti-VEGF agents (40). We used a Bruker timsTOF™ Pro mass spectrometer to analyze the VH. This instrument couples trapped ion mobility spectrometry (TIMS) to high-resolution time-of-flight MS. Use of the ion mobility parameter added a dimension of separation and increased overall system peak capacity in the gas phase. Ultimately, this resulted in better coverage of the proteome (41, 42).

However, there were some limitations to this study. First, the sample size of each group was small. Second, we did not conduct in-depth research on the results of this experiment at the animal or cell level. Large-scale clinical studies and *in vitro* experiments are necessary to investigate the molecular mechanisms of IVR in PDR. At the same time, we should also study the therapeutic effects of other anti-VEGF drugs on PDR in further study.

## Conclusions

In conclusion, this study demonstrated that VH protein profiles differed in response to ranibizumab treatment. Proteins that showed increased expression after IVR treatment were significantly enriched in “GnRH secretion” and “Circadian rhythm” pathway. This report reveals IVR treatment may protect against PDR by promoting SPP1 expression through “GnRH secretion” and “Circadian rhythm” signaling pathway, providing a new perspective on the mechanism of ranibizumab treatment to PDR.

## Data Availability Statement

The datasets presented in this study can be found in online repositories. The names of the repository/repositories and accession number(s) can be found below. The mass spectrometry proteomics data have been deposited to the ProteomeXchange Consortium via the PRIDE (http://www.ebi.ac.uk/pride) partner repository with the dataset identifier PXD027592; Username: reviewer_pxd027592@ebi.ac.uk; Password: b7I8VHZMs.

## Ethics Statement

The studies involving human participants were reviewed and approved by Ethics Committee of Shanghai General Hospital (Ethical approval number: 2021KY031). The patients/participants provided their written informed consent to participate in this study. Written informed consent was obtained from the individual(s) for the publication of any potentially identifiable images or data included in this article.

## Author Contributions

XS and ZZ: Conceptualization. XS and CZ: Formal analysis. XS: Methodology, data curation, and writing original draft. ZZ: Writing review and editing. All authors read and approved the final manuscript.

## Funding

Research reported in this publication was supported by the Program of the National Natural Science Foundation of China (81770947); National Key R&D Program of China (2016YFC0904800,2019YFC0840607; National Science and Technology Major Project of China (2017ZX09304010).

## Conflict of Interest

The authors declare that the research was conducted in the absence of any commercial or financial relationships that could be construed as a potential conflict of interest.

## Publisher's Note

All claims expressed in this article are solely those of the authors and do not necessarily represent those of their affiliated organizations, or those of the publisher, the editors and the reviewers. Any product that may be evaluated in this article, or claim that may be made by its manufacturer, is not guaranteed or endorsed by the publisher.

## Supplementary Material

Supplementary Figure S1 Protein overlap analysis in the pre group and post group.

Supplementary Figure S2 Targeted peptide PRM_ Skyline analysis results.

Supplementary Table S1 Protein identification and quantification, a total of 2680 vitreous proteins were identified.

Supplementary Table S2 A total of 1874 GO terms related to all 38 differentially expressed proteins were identified.

Supplementary Table S3 Information of peptide, precursor Mz, fragment ion, areas and other original data for PRM quantitative Skyline data.

Supplementary Table S4 Fold change of protein level in Label-free and PRM (Post / Pre).

## Abbreviations

DR: Diabetic retinopathy
PDR: Proliferative DR
IVR: Intravitreal injection of ranibizumab
VH: Vitreous humor
PRM: Parallel reaction monitoring
PPV: Pars plana vitrectomy
GO: Gene ontology
KEGG: Kyoto Encyclopedia of Genes and Genomes
PPI: Protein–protein interaction
FC: Fold change
BP: Biological processes
MF: Molecular functions
CC: Cellular components
TIMS: trapped ion mobility spectrometry.
