# Supplementary figures and images for: Differences in Vitreous Protein Profiles in Patients With Proliferative Diabetic Retinopathy Before and After Ranibizumab Treatment
Source: Front Med (Lausanne). 2022 May 27;9:776855. doi: 10.3389/fmed.2022.776855 (PMC9198965; doi:10.3389/fmed.2022.776855)

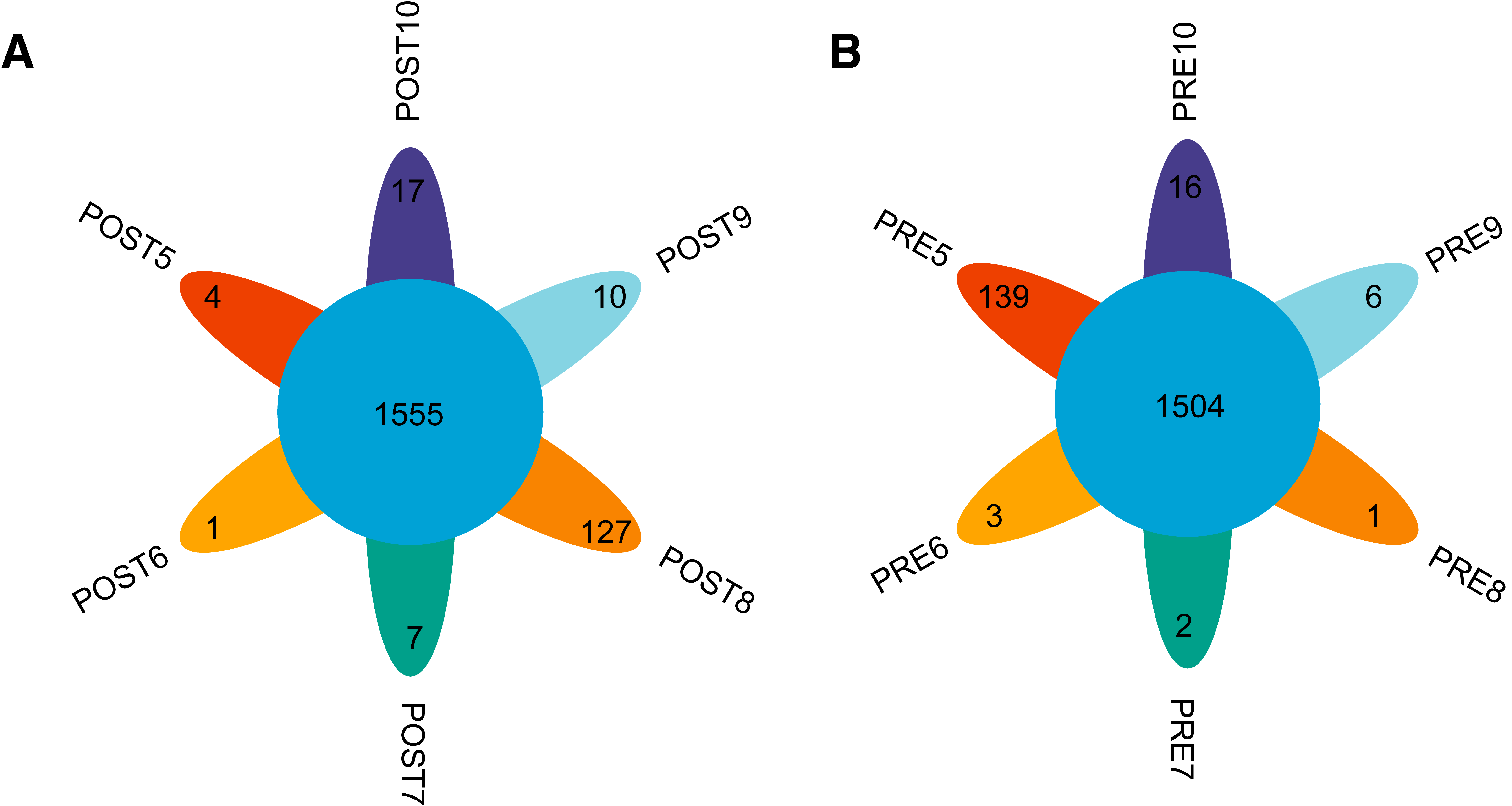

Supplement: Supplementary Figure S1 — Protein overlap analysis in the pre group and post group. [file Image_1.TIF]
