# Supplementary material for: Differences in Vitreous Protein Profiles in Patients With Proliferative Diabetic Retinopathy Before and After Ranibizumab Treatment
Source: Front Med (Lausanne). 2022 May 27;9:776855. doi: 10.3389/fmed.2022.776855 (PMC9198965; doi:10.3389/fmed.2022.776855)

# Supplementary Materials

**Supplementary Fig S1 Protein overlap of samples in the post group and the pre group.**


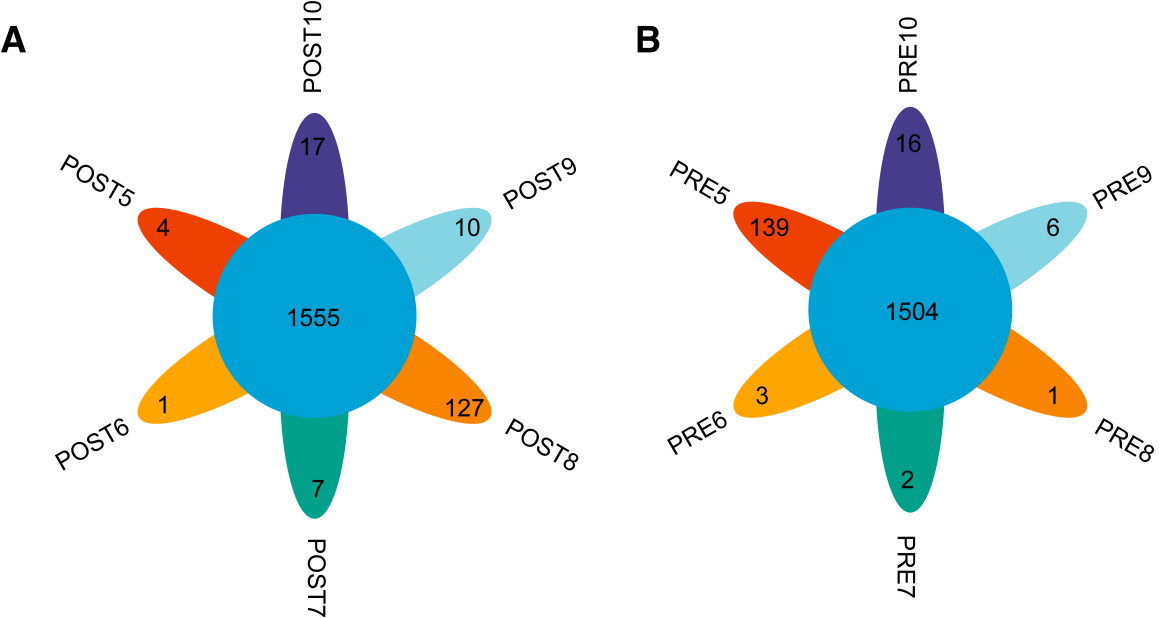


Fig S1 Protein overlap of samples in the post group and the pre group. (A) VH samples collected from 6 PDR patients before IVR treatment were used as the pre group. (B) VH samples collected from the same 6 PDR patients three days after IVR treatment were used as the post group.

**Supplementary Fig S2 Targeted peptide PRM_ Skyline analysis results**

1. Protein: P01764, IGHV3-23

Peptide sequence: AEDTAVYYCAK


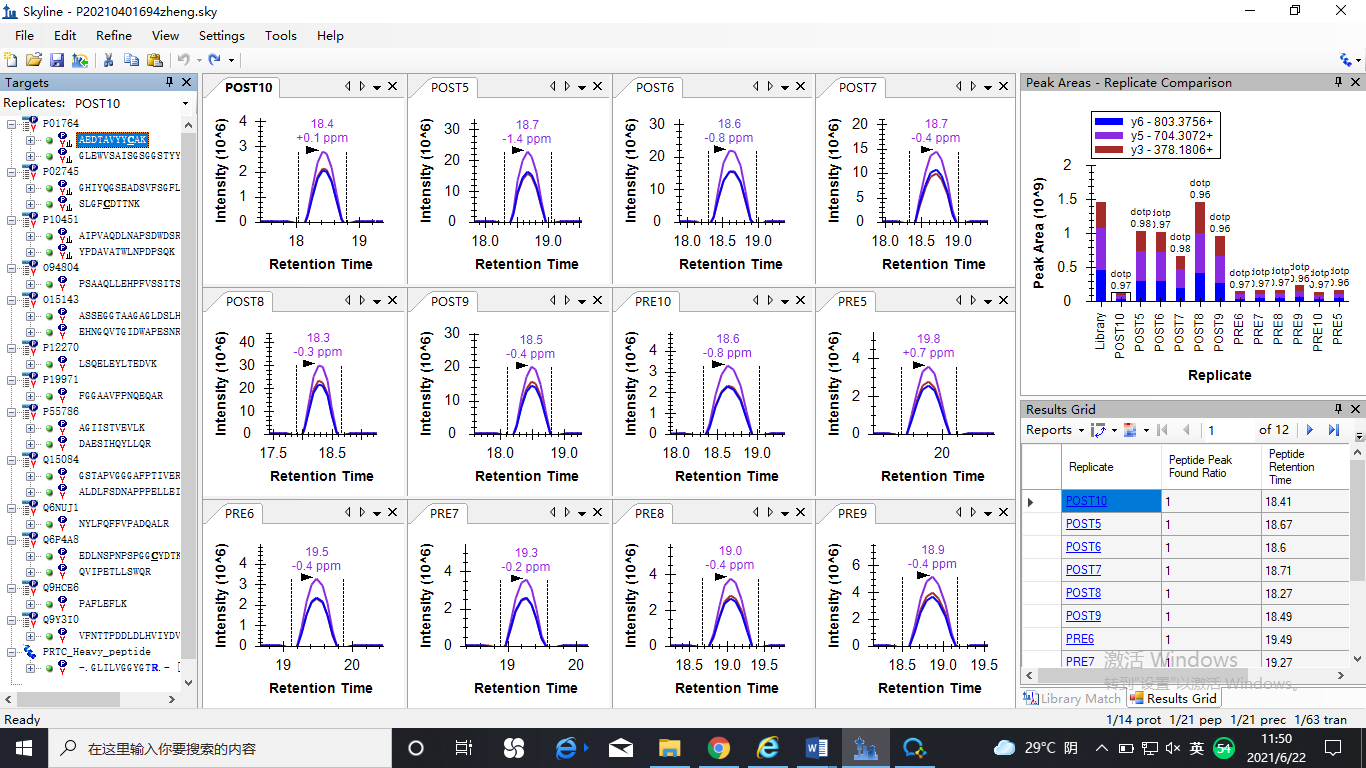

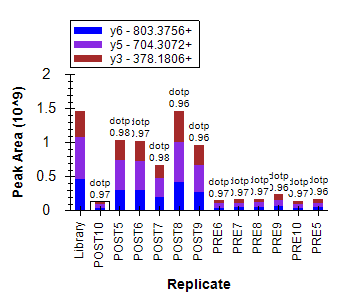


2. Protein: Q9Y3I0, RTCB

Peptide sequence: VFNTTPDDLDLHVIYDVSHNIAK


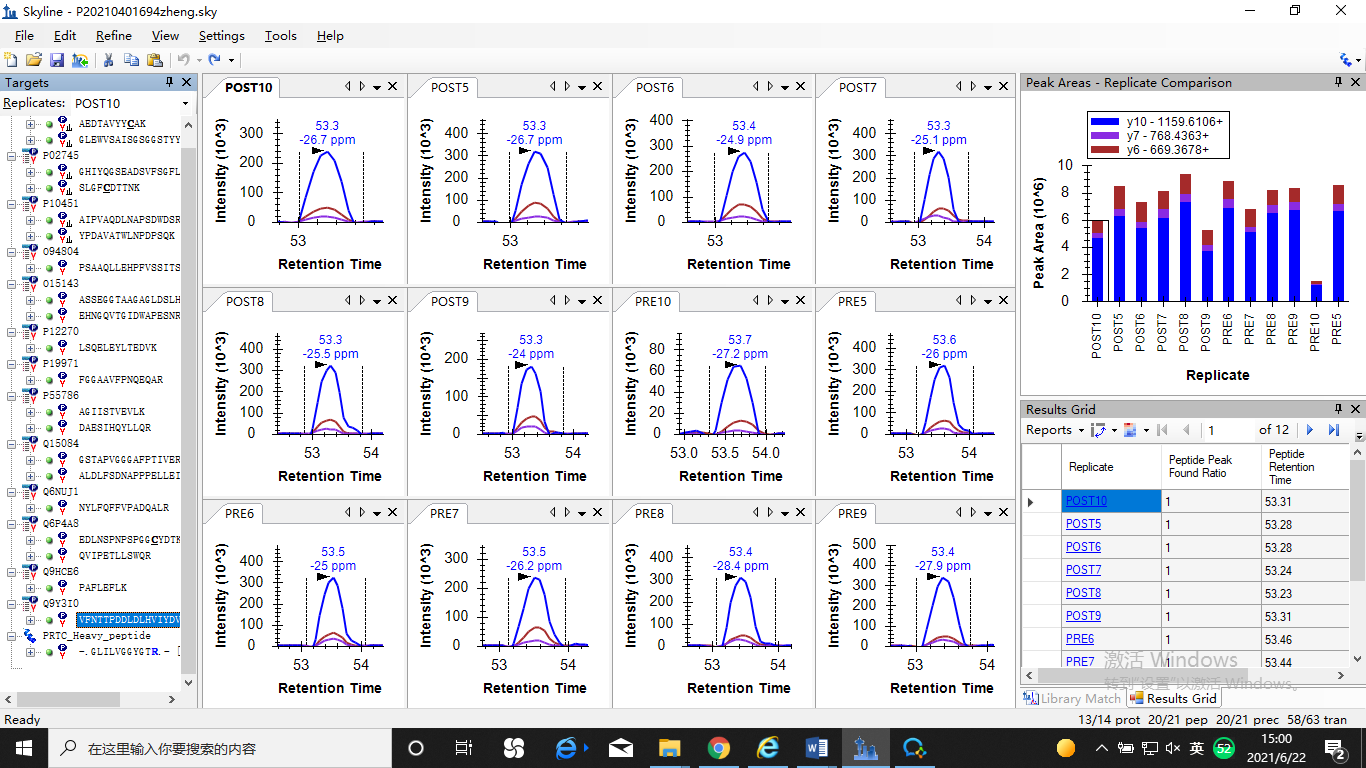


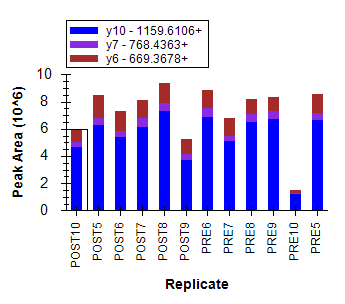


3. Protein: P10451, SPP1

Peptide sequence: AIPVAQDLNAPSDWDSR


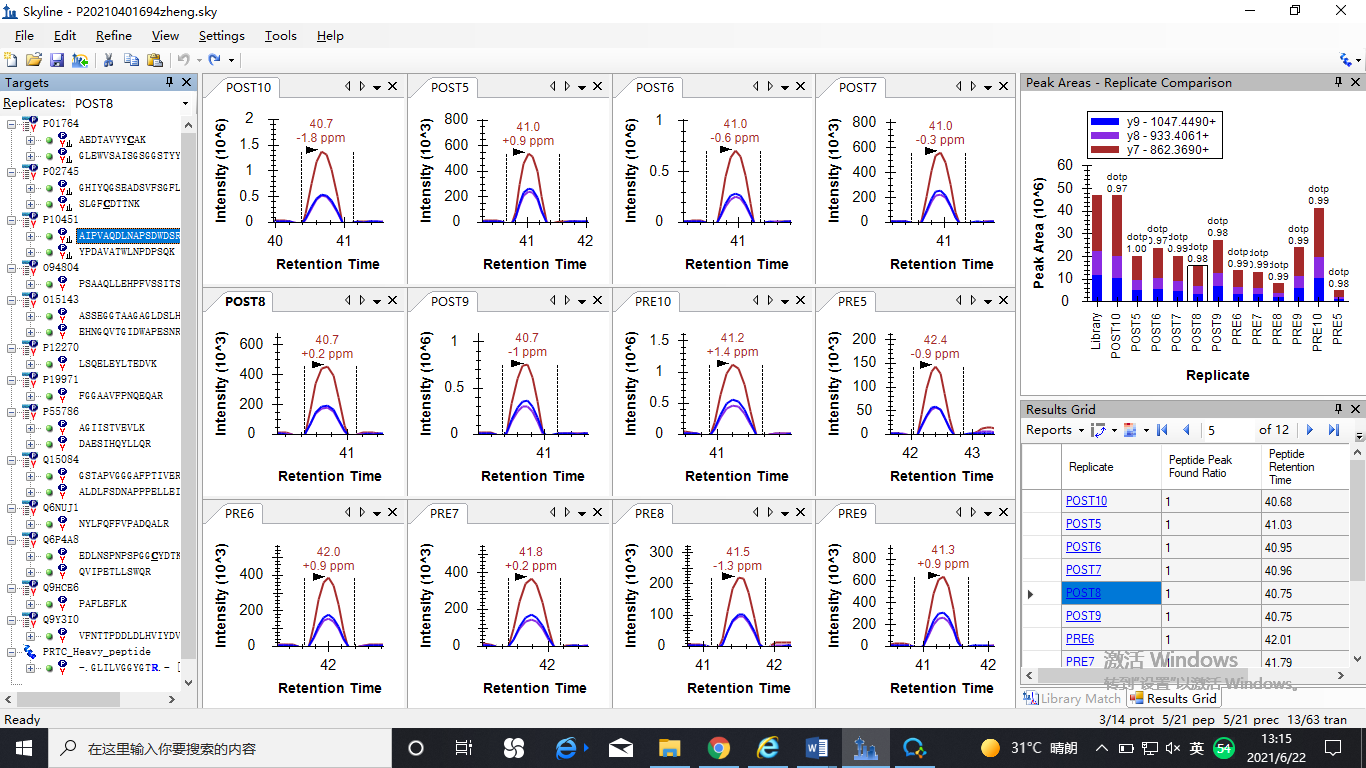


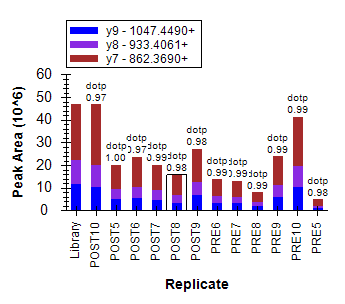


4. Protein: P19971, TYMP

Peptide sequence: FGGAAVFPNQEQAR


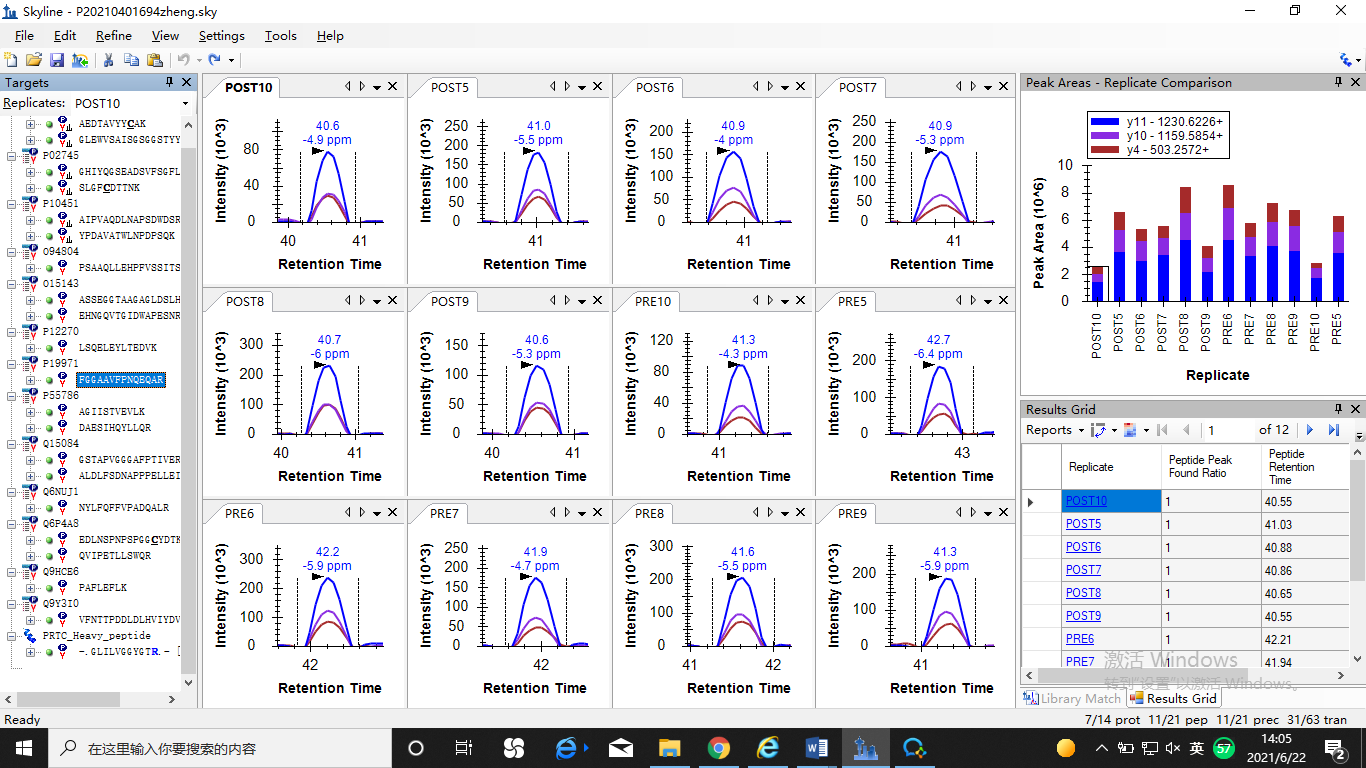


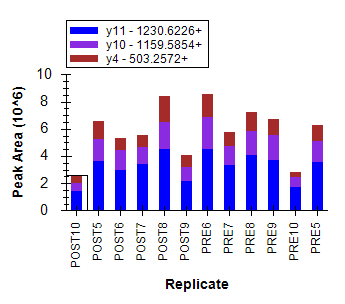


5. Protein: Q6NUJ1, PSAPL1

Peptide sequence: NYLFQFFVPADQALR


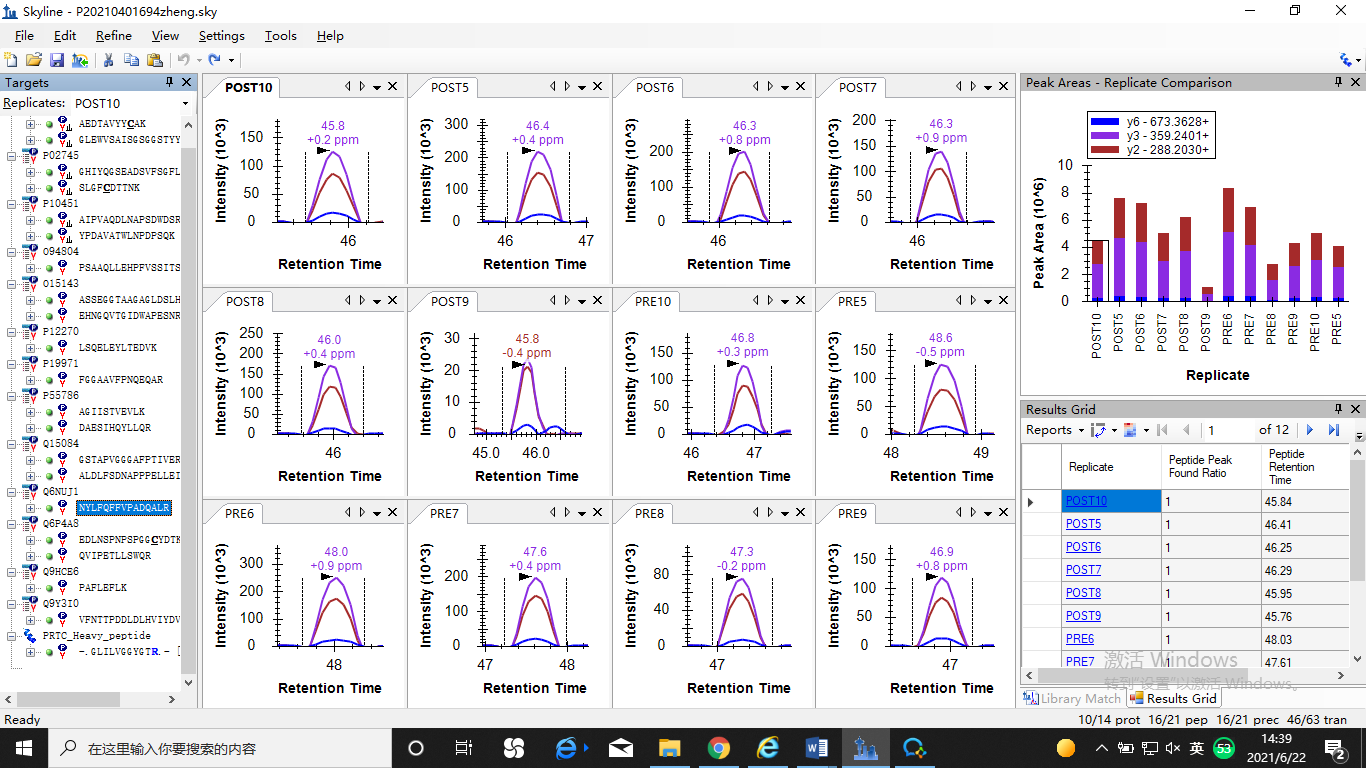


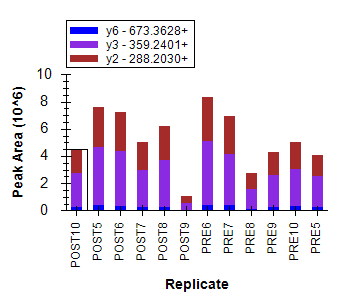


6. Protein: P55786, NPEPPS

Peptide sequence: AGIISTVEVLK


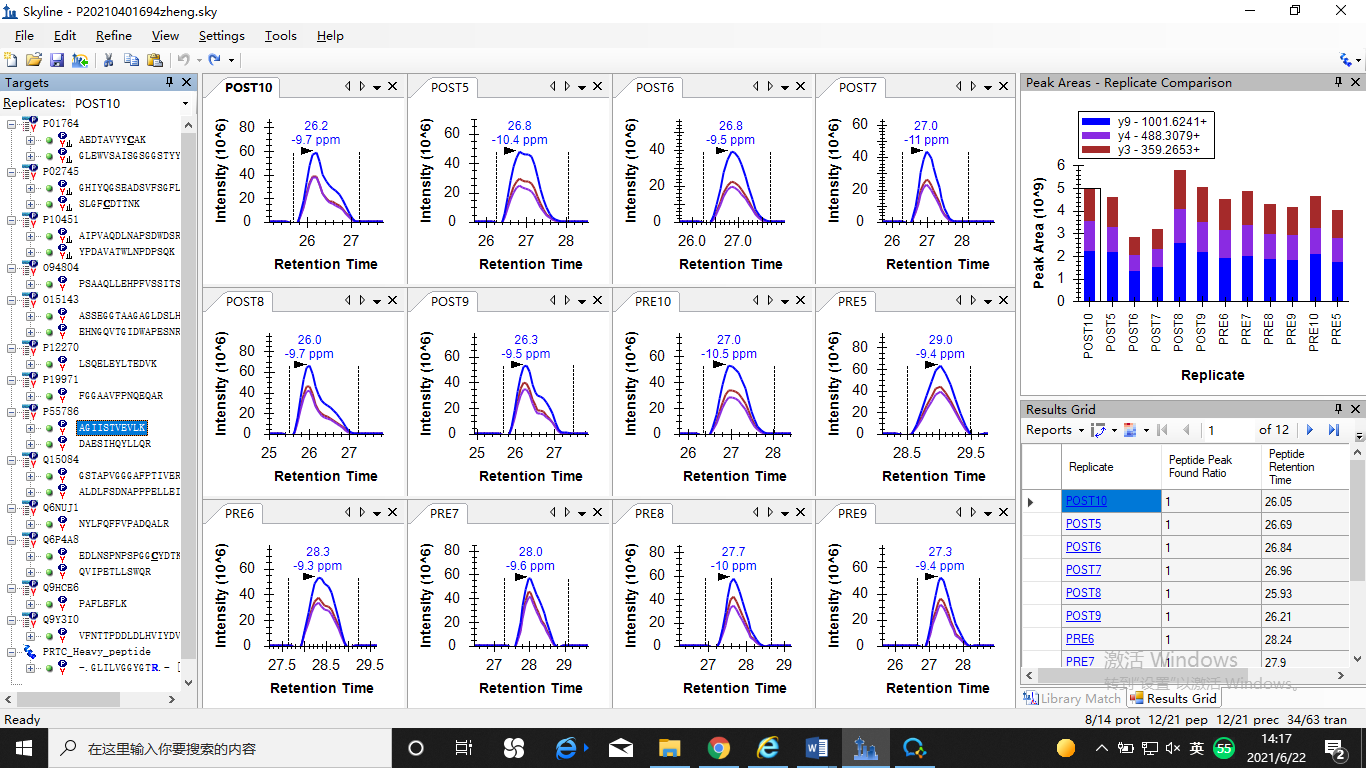


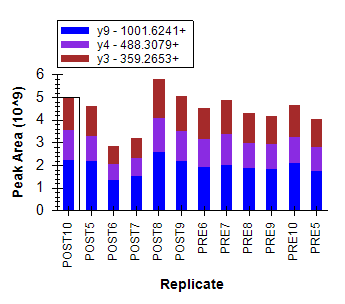


7. Protein: P02745, C1QA

Peptide sequence: SLGFCDTTNK


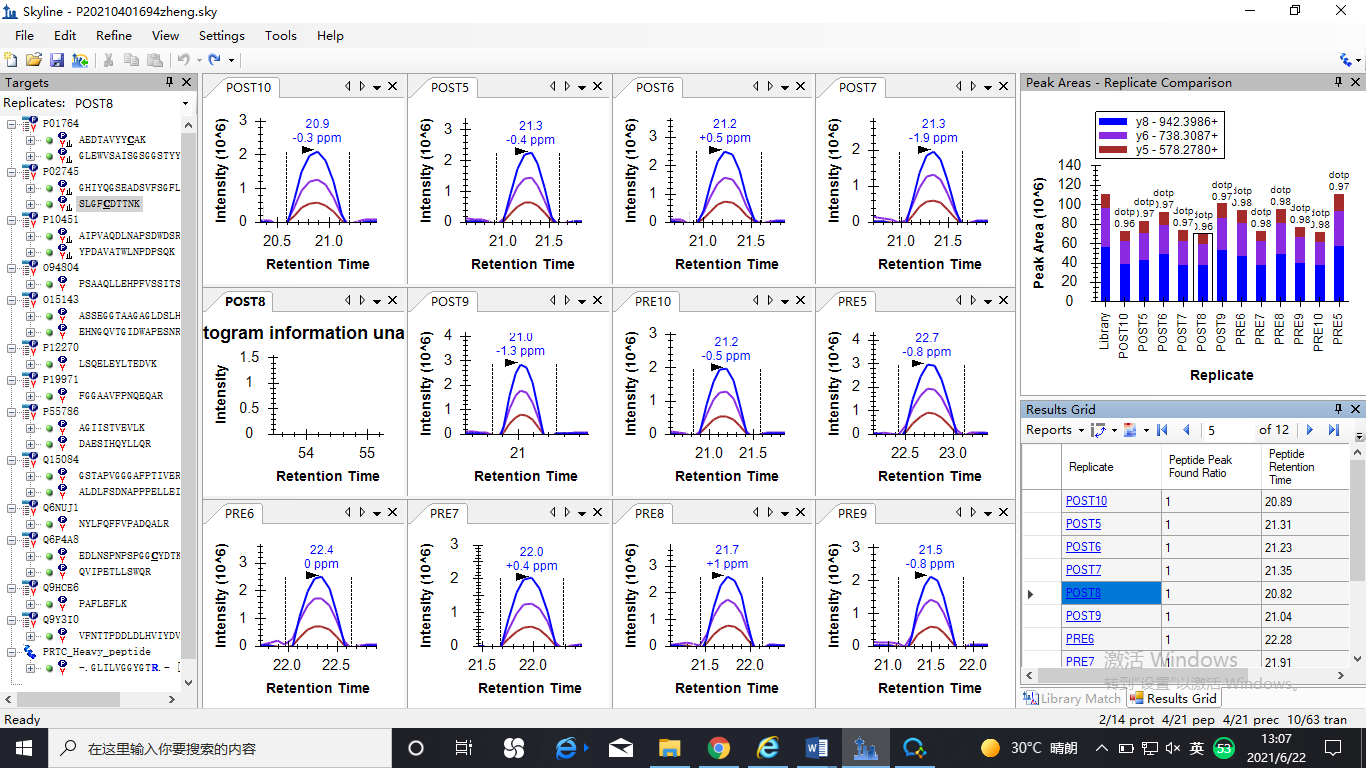


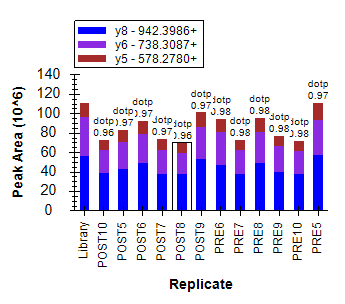

Supplement: Supplementary Figure S2 — Targeted peptide PRM_ Skyline analysis results. [file Data_Sheet_1.DOCX]
